# Quantitative analysis of gaze and body movement differences by proficiency in direct and video laryngoscope intubation

**DOI:** 10.1038/s41598-026-44432-5

**Published:** 2026-04-02

**Authors:** Yukinori Yasuda, Shoichiro Takehara, Soichiro Inoue

**Affiliations:** 1 Graduate School of Science and Technology, Sophia University, Tokyo, 102-8554 Japan; 2 Department of Engineering and Applied Sciences Faculty of Science and Technology, Sophia University, Tokyo, 102-8554 Japan; 3 Department of Anesthesiology, St. Marianna University School of Medicine, Kanagawa, 216-8511 Japan

**Keywords:** Diseases, Health care, Medical research

## Abstract

In the practice of medicine, tracheal intubation is a critical procedure, yet medical education often lacks quantitative instructional methods, relying instead on intuitive guidance from experts. This study used motion capture and eye tracking to analyze differences in movement and gaze between novices and experts during tracheal intubation. It also compared the use of direct and video laryngoscopes. Results showed that with a direct laryngoscope, experts directed their gaze farther than novices during the laryngoscope insertion phase, with a significant difference in eye-to-gaze distance [experts: 0.465 m; novices: 0.325 m; t (12) = 2.690, *p* = 0.020]. No significant difference was found when a video laryngoscope was used, suggesting that novices could perform expert gaze behavior. Head movement analysis revealed that experts maintained a greater distance from the mannequin’s mouth when using direct laryngoscopes (experts: 0.529 m; novices: 0.331 m). With video laryngoscopes, the gap narrowed (experts: 0.550 m; novices: 0.457 m). A t-test comparing novice head-to-mouth distance between device types showed a significant difference [t (14) = 4.355, *p* = 0.001]. These findings highlight how video laryngoscopes may help novices approximate expert behavior, offering potential for improved training methods.

**Supplementary Information:**

The online version contains supplementary material available at 10.1038/s41598-026-44432-5.

In medical practice, a wide range of procedures are performed on patients, including those required in medical emergencies to provide life-saving care. These medical procedures range from basic tasks, such as injections, suturing, and operation of intravenous infusion pumps, to emergency resuscitation procedures, including tracheal intubation and intravenous cannulation^1–3^. Tracheal intubation is particularly critical because first-pass success reduces airway-related complications, and prolonged intubation time increases the risk of hypoxemia^4^. Medical procedures are highly complex and sometimes medical errors occur, causing injuries or sequelae to patients due to human mistakes made by physicians with inadequate skills^5,6^. Consequently, education in medical settings is crucial. In fact, modern medical education has traditionally relied on teaching with materials like textbooks and videos, as well as simulation-based practical training using human body mannequins. However, practical training often relies on intuitive guidance from experienced physicians, making it difficult for novice physicians to grasp proper instrument handling and application of force^7^. Therefore, improving the educational environment for physicians by making efforts to accurately and efficiently transmit medical skills to novice physicians can reduce medical errors.

In this study, we investigated differences in medical procedure proficiency from an engineering perspective. Previous research in the medical field has examined medical procedures by analyzing muscle activity using biosignals such as electromyography^8^. However, the accuracy of medical procedures is influenced by human behavior, and biosignal analysis focuses on the physiological responses that occur after the action has been performed. As a result, this approach has limitations in fully elucidating the entire sequence of movements, which directly affects the improvement of medical procedures. In contrast, the engineering field has conducted quantitative analyses of human body movement and gaze movement in human performance studies. These studies, which used motion capture systems and eye-tracking devices, have enabled the visualization of physical movements, and they have contributed to improved skill and proficiency. For example, in the field of sports science, research has been conducted to visualize differences between experts and novices using three-dimensional motion data. Monika et al. demonstrated that, during karate kicking motions, there are significant differences in the movements of the head, trunk, hip, knee, and ankle^9^. Furthermore, in the field of traditional performing arts, research has been conducted to analyze the differences between experts and novices, with the aim of accelerating skill acquisition. The study by Sato et al. suggested that the use of motion capture in dance acquisition enables novices to observe their movements through objective data, thereby facilitating awareness of specific issues and allowing for efficient skill acquisition in a short time^10^. Furthermore, in the construction industry, Morita et al. revealed differences in visual attention through eye-tracking analysis, confirming that experts tend to observe the entire site, whereas novices fail to focus on the appropriate areas^11^. These examples make it clear that, in fields such as body culture and industry, conducting motion and gaze measurements enables efficient skill acquisition based on objective data.

In this study, motion measurement using motion capture and gaze measurement using an eye-tracking device were combined, and we examined differences in body movement and gaze movement according to skill level, focusing on one of the many medical procedures: tracheal intubation. Tracheal intubation is performed by anesthesiologists and paramedics in situations such as anesthesia and emergency medicine, with the aim of managing the airway of patients with dyspnea or a compromised airway^12,13^. The reliability of airway management through this procedure is high, and it is considered the first-choice method when long-term airway maintenance or mechanical ventilation requiring high airway pressure is necessary^14^. Furthermore, because this procedure generates aerosols, it is necessary to perform the procedure while implementing infection prevention measures when treating patients with infectious diseases^15^. Therefore, the difficulty of mastering this procedure is considered high, and differences in proficiency are said to be significant. Based on these considerations, this procedure was selected as the subject of this study.

Tracheal intubation is performed by securing the visual field using a medical device called a laryngoscope and inserting a tracheal tube through the mouth or nose via the larynx. During this process, it is necessary to flexibly adjust the application of force and the handling of instruments depending on the patient’s body size, the patient’s skeletal structure, and the type of laryngoscope used. The types of laryngoscopes are broadly classified into two categories: direct laryngoscopes and video laryngoscopes. A direct laryngoscope allows the operator to view the tracheal opening or glottic opening directly from outside the mouth, whereas a video laryngoscope has a camera at the tip of the blade that enables indirect visualization of the larynx on a monitor^16,17^. In recent years, the use of video laryngoscopes has been increasingly promoted over direct laryngoscopes in medical education settings, and several studies have reported improved success rates in procedures using video laryngoscopes^18^; however, the reasons for this improvement have not been explained with quantitative indicators. A series of studies conducted by Hamabe and colleagues, as well as other researchers, used motion capture systems and eye-tracking systems to investigate gaze and body movements during intubation procedures^19–21^. However, to the best of our knowledge, no studies have investigated how differences in laryngoscope type affect gaze movement and body behavior.

Based on these considerations, this study attempted to clarify the differences in the characteristics of gaze movement and body movement during the use of direct laryngoscopes and video laryngoscopes, as well as those arising from various levels of operator proficiency, through simultaneous measurement and integrative analysis of eye-tracking and body motion data. Through this multifaceted approach, the study quantitatively identified the relationship between gaze behavior and body movement—an aspect not addressed in previous research. It is anticipated that these findings will contribute to improvements in medical education environments and, consequently, to the advancement of medical skills.

## Methods

### Experimental setup

**Fig. 1:**
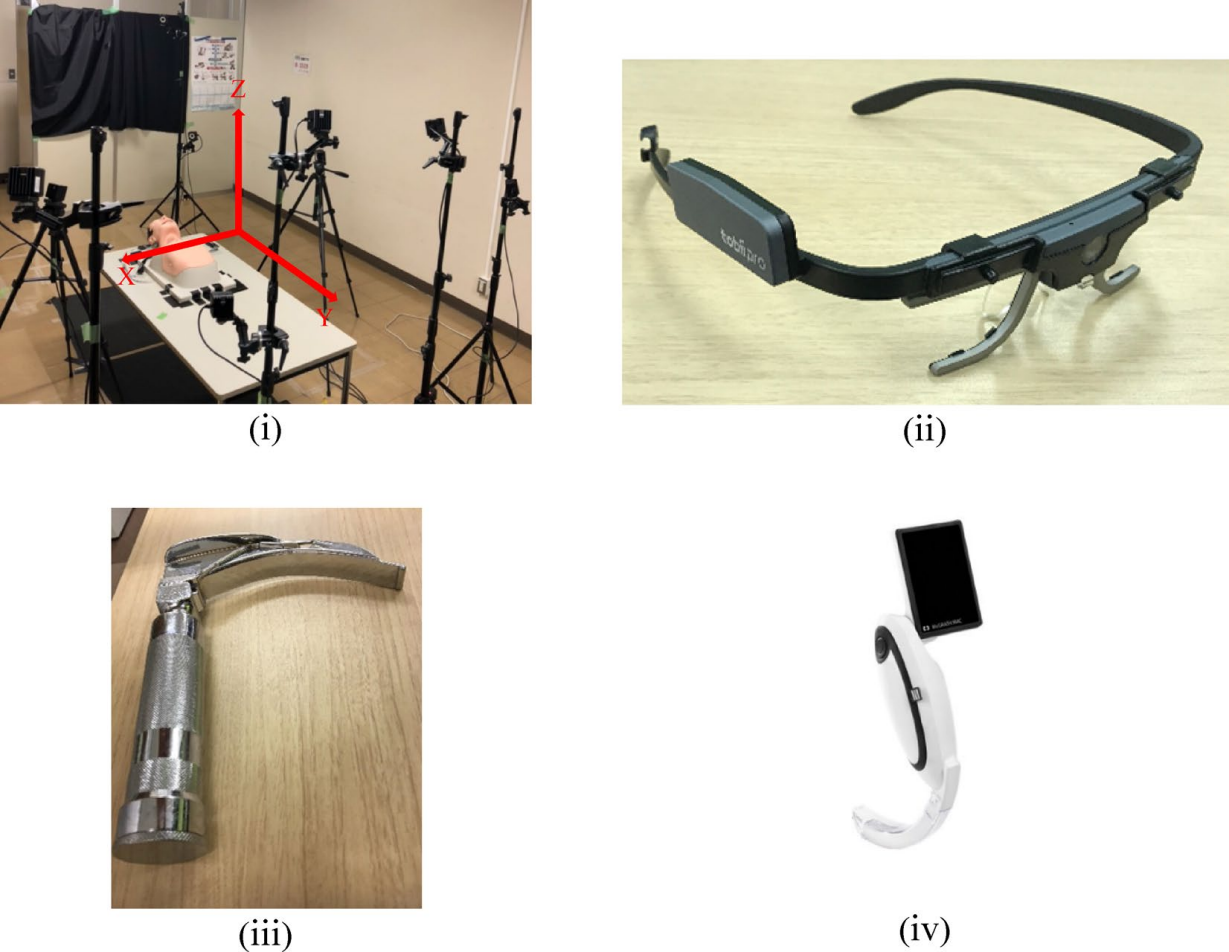
Experimental environment and devices used in this study: (i) Experimental environment, (ii) Tobii Pro Glasses 2, (iii) Direct laryngoscope, and (iv) Video laryngoscope^25^.

An overview of the experimental setup is shown in Fig. 1 (i). Twelve motion capture cameras were arranged around a surgical table and the experimental subject. The surgical table was placed at the center of the area enclosed by the cameras. A laryngoscope was placed on the left side of the surgical table and a tracheal tube on the right side from the perspective of the experimental subject. To standardize the experimental conditions in this experiment, the height of the table was kept constant throughout all trials. Specifically, the height was adjusted so that the mouth of the mannequin was positioned approximately 0.85 m above the ground, which corresponds roughly to the waist level of the subjects. In addition, de Laveaga et al. confirmed that differences in table height do not cause differences in procedure time or failure rate^22^. Therefore, we assumed that table height does not affect the procedure, and thus the table was kept at a constant height in all trials.

### Motion capture camera

Ten Prime13 cameras and 2 Prime13W cameras manufactured by OptiTrack were used in this experiment. The resolution of these cameras is 240 fps, and both models are capable of capturing images at 1.3 megapixels. Prime13, which uses a standard lens, has a horizontal field of view of 56 deg, resulting in a visible area 3.19 m wide × 2.55 m tall at a distance of 3 m. Prime13W, which uses a wide-angle lens, has a horizontal field of view of 82 deg, resulting in a visible area 5.22 m wide × 4.20 m tall at a distance of 3 m, allowing for wide-area capture. In this motion capture system, the coordinates of a single marker cannot be determined unless it is simultaneously captured by at least two cameras. Therefore, the cameras were arranged to surround the experimental subject in a manner that ensured that all markers were captured, and measurements were conducted accordingly.

### Eye-tracking device

Eye gaze measurements were conducted using the Tobii Pro Glasses 2 manufactured by Tobii Technology Inc., as shown in Fig. 1 (ii). This device measures visual information such as pupil position, gaze direction, and focal point by irradiating the eye cornea with near-infrared light from the measurement device based on the corneal reflection method, and analyzing eye movements through video analysis^23^.

### Types of laryngoscopes

There are various types of laryngoscopes^24^. Direct laryngoscopes and video laryngoscopes are mainly used in medical situations, and both of these types of laryngoscopes were used in this experiment. Specifically, among the direct laryngoscopes, the Macintosh type, which features a curved scope insertion blade, was used. For the video laryngoscope, the McGRATH model was used. Both laryngoscopes are shown in Fig. 1 (iii) (iv). The Macintosh type measures 297 mm in total length and 28 mm in width, whereas the McGRATH model has a total length of 180 mm and a width of 68 mm^25^. Details of the laryngoscopes are provided in the supplementary materials.

**Medical training mannequin.** For this study, the DAM Simulator Training Model produced by Kyoto Kagaku Co., Ltd., was employed. This anatomical mannequin is widely used in medical education for hands-on training for tracheal intubation^26^.

**Fig. 2:**
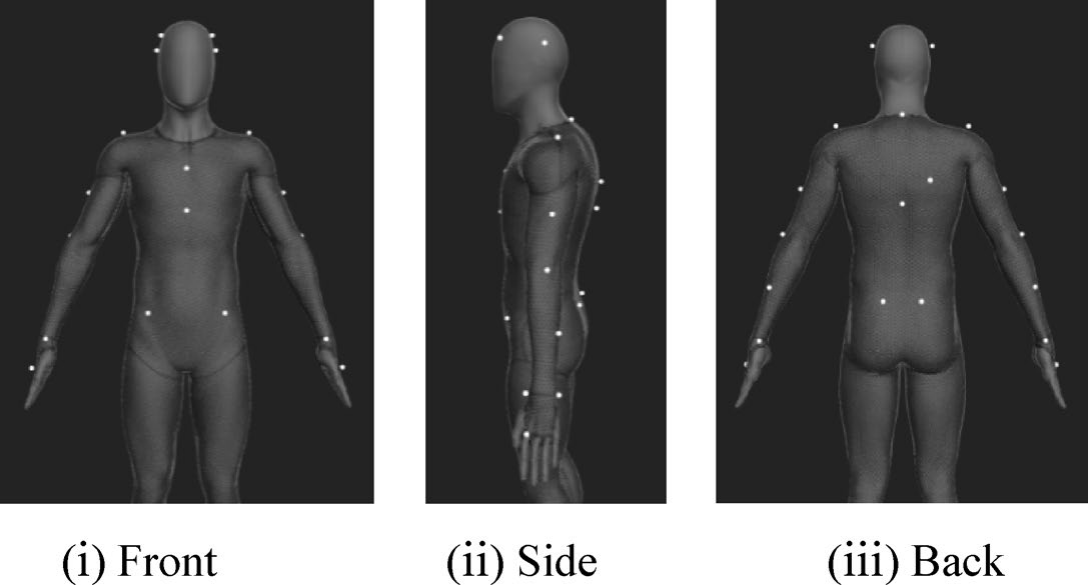
Marker placement locations on the experimental subjects. (i) Front, (ii) Side, (iii) Back.

**Positioning of markers.** The marker placement locations on each experimental subject are shown in Fig. 2. A total of 27 markers were attached: 4 on the head, 12 on the arms, 2 on the shoulders, 2 on the back, 2 on the neck, 1 on the chest, and 4 on the waist.

### Subject information

The experiment was conducted with the cooperation of 15 subjects. Details for the subjects and experimental procedures are given below. This study was approved by the Ethics Committee for Research Involving Human Subjects at Sophia University, and informed consent was obtained from all participants prior to their involvement. All methods were carried out in accordance with relevant guidelines and regulations.

Regarding the subjects, individuals who had performed tracheal intubation more than 100 times and held appropriate qualifications as anesthesiologists were defined as experts, while those who had performed fewer than 30 tracheal intubations were defined as novices. Among the total of 15 participants involved in this experiment, seven were experts and eight were novices. Information about each subject is provided in the supplementary materials. Previous studies using motion capture have been conducted with approximately seven participants; therefore, we examined seven experts and eight novices in the present study^27^, ^28^. In this experiment, subjects performed the procedure without wearing contact lenses or glasses. However, regarding the visual acuity of each participant, because the eye-tracking device is equipped with corrective lenses, calibration was performed according to the visual condition of their usual daily life, and the experiment was started accordingly. Therefore, the visual acuity of each participant was taken into consideration.

### Statistical analysis

Statistical analysis was conducted using the t-test. The significance level was set at the commonly used threshold of *p* < 0.05.

## Experimental procedure

The experiment was conducted as follows:

1. Each subject signed a consent form after agreeing to the purpose and content of the experiment.
2. Subjects donned a motion capture suit, and markers were attached to their bodies.
3. To identify the marker positions, subjects were instructed to stand in a specified posture, and a 3D computer-generated model was created.
4. Pupil calibration was performed for each subject.
5. Subjects moved to the initial position and performed tracheal intubation 10 times for measurement.
6. If any data were missing, the measurements were repeated until 10 complete sets of data were obtained.

## Results

### Target areas and planes of analysis

We present the measurement results of gaze and body movements when using direct and video laryngoscopes. This study investigated the relationship between visual attention and physical movement during tracheal intubation procedures, in accordance with differences in operator proficiency. In tracheal intubation procedures, the operator is aware of movements in the sagittal plane^29^. Therefore, we conducted an analysis focusing on the depth and vertical directions, which correspond to the sagittal plane. In this paper, the depth direction is defined as the Y axis and the vertical direction as the Z axis; thus, the Y-Z plane represents the sagittal plane. Additionally, body movement analysis was performed with a focus on the head, which is considered to be related to gaze shifts^30^.

### Eye movement

**Fig. 3:**
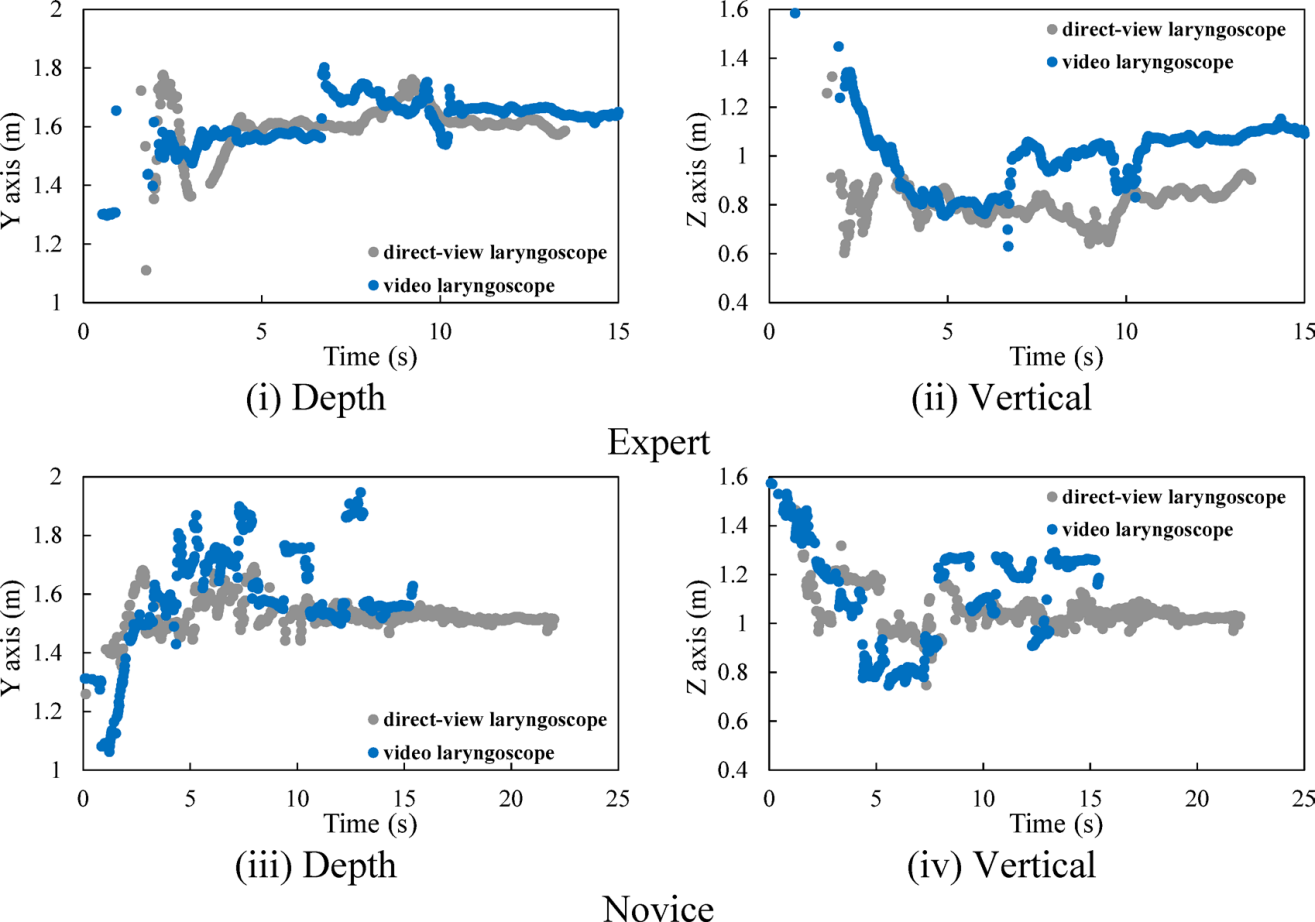
Time history of gaze points.

**Fig. 4:**
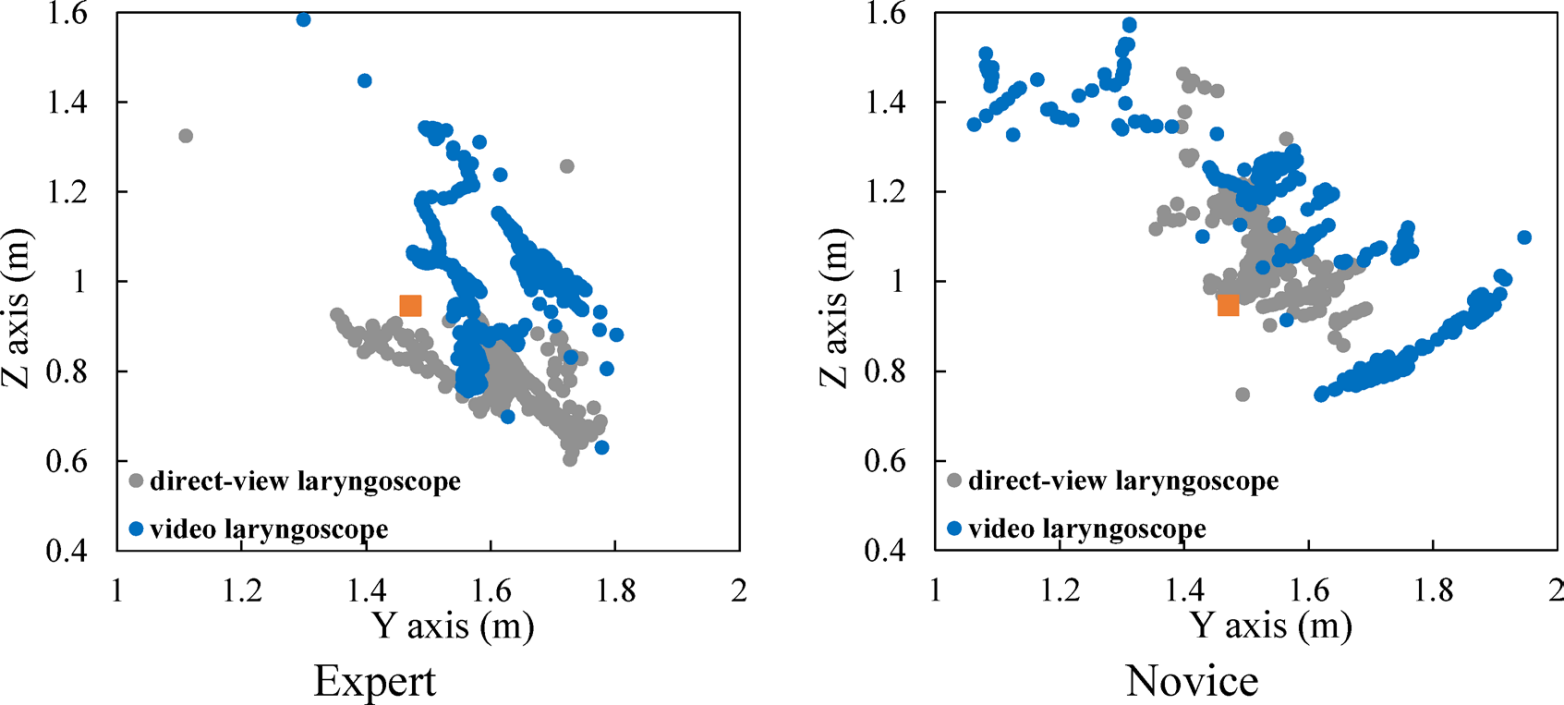
Gaze point distribution in the sagittal plane.

Figure 3 shows an example of the time history of gaze in the depth and vertical directions for both experts and novices, and Fig. 4 illustrates the distribution of gaze fixation points on the sagittal plane. The orange square in Fig. 4 indicates the position of the mouth of the human body mannequin. First, we describe the gaze behavior when using a direct laryngoscope. At the beginning of the recording, the subjects assumed a T-pose with both arms extended horizontally to ensure accurate recognition of the markers. This posture allows for stable acquisition of marker position data in the initial state. In the analysis of the treatment motion, the focus was placed on the movements starting from the moment the T-pose was terminated and the actual procedure began. The procedure start time was defined as T = 4.0 s for the expert and T = 3.0 s for the novice. We begin by describing the gaze behavior of experts. Figure 3 (i) shows that, in the depth direction, the gaze points of the experts are concentrated around 1.6 m and distributed within the range from 1.5 m to 1.7 m. Additionally, from Fig. 3 (ii), it is evident that, in the vertical direction, the gaze points are mainly distributed below 1.0 m. As shown in Fig. 4, in the sagittal plane, the distribution of gaze points is located anteriorly and inferiorly compared to the position of the mouth of the human model. Comparable patterns were observed in other experts. We describe the gaze behavior of novices. According to Fig. 3 (iii), the gaze points of the novices are concentrated around 1.5 m and distributed within the range from 1.4 m to 1.7 m in the depth direction. In addition, as shown in Fig. 3 (iv), their gaze points are distributed over a range of around 1.0 m in the vertical direction. From Fig. 4, it can be seen that, in the sagittal plane, many of the gaze points in the distribution are located posteriorly and superiorly compared to the position of the mouth of the human mannequin. Comparable patterns were observed in other novices.

We describe the gaze behavior when a video laryngoscope is used. The treatment start time was defined as T = 4.0 s for the expert and T = 2.0 s for the novice. We begin by describing the gaze behavior of experts. According to Fig. 3 (i), in the depth direction, their gaze points are maintained at 1.55 m until 7.0 s, after which the gaze distribution becomes concentrated around 1.65 m and extends over the range from 1.5 m to 1.8 m. Furthermore, as shown in Fig. 3 (ii), in the vertical direction, the gaze is maintained below 1.0 m until 7.0 s, after which the gaze distribution becomes concentrated around 1.0 m. From Fig. 4, the distribution of gaze points, when compared to the position of the mouth of the human mannequin, exhibits two distinct concentrations: one in the forward-downward direction and the other in the forward-upward direction. This shows that, during the initial phase of the procedure, the expert primarily directs attention toward the mouth of the human mannequin, whereas in the subsequent phase, attention is focused on the monitor screen of the video laryngoscope. We describe the gaze behavior of novices as follows. According to Fig. 3 (iii), their gaze points in the depth direction are distributed within the range from 1.5 m to 1.8 m until 7.5 s, after which they are concentrated around 1.55 m. Furthermore, as shown in Fig. 3 (iv), their gaze points in the vertical direction are below 1.0 m until 7.5 s, after which they are distributed within the range from 1.0 m to 1.3 m. From Fig. 4, it can be seen that, in the sagittal plane, compared to the position of the mouth of the human mannequin, the distribution is widely spread forward and downward, and also distributed forward and upward. This shows that, during the first half of the procedure, attention is directed toward the mouth of the human mannequin, whereas during the latter half, the gaze is also directed toward the monitor screen of the video laryngoscope.

### Head movement

**Fig. 5:**
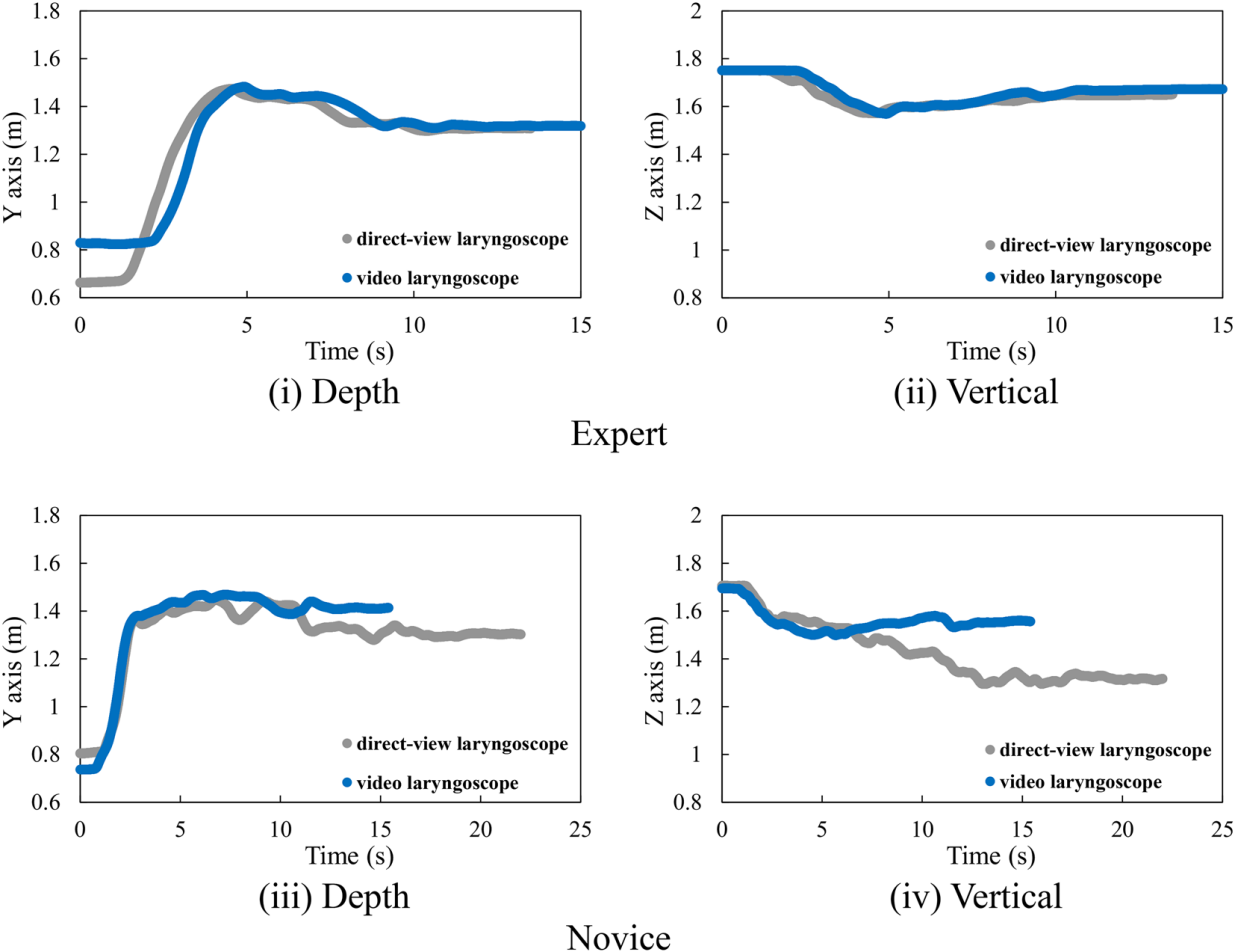
*Time history of head movement*. (iii) Depth, (iv) Vertical.

**Fig. 6:**
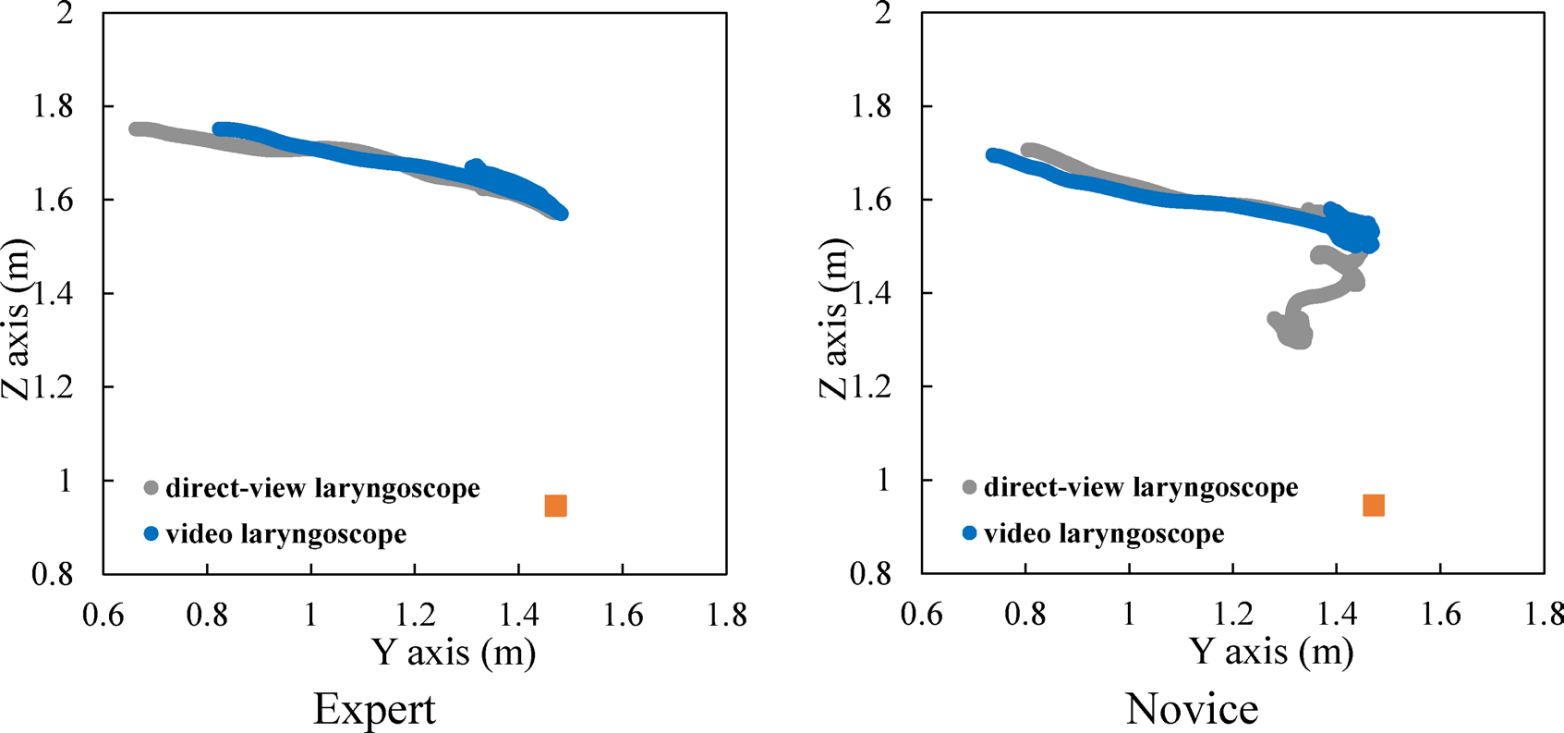
Head position trajectory in the sagittal plane.

Figure 5 shows an example of the time history of head movement in the depth and vertical directions for experts and novices, and Fig. 6 presents the trajectory of the head in the sagittal plane. The orange square in Fig. 6 indicates the position of the mouth of the human body mannequin.

First, the characteristics of using a direct laryngoscope are described. Initially, the head movements of experts are presented. According to Fig. 5 (i), the displacement in the depth direction initially increases, and movement occurs within the range of 1.3 m to 1.4 m. Furthermore, as shown in Fig. 5 (ii), the displacement in the vertical direction initially decreases, and movement occurs within the range of 1.6 m to 1.7 m. From Fig. 6, it can be observed that the trajectory of the head in the sagittal plane is linear. Comparable patterns were observed in other experts.

Next, the head movements of novices are presented. According to Fig. 5 (iii), the displacement in the depth direction initially increases, and movement occurs within the range of 1.4 m to 1.5 m. In addition, as shown in Fig. 5 (iv), the displacement in the vertical direction initially decreases, and movement occurs within the range of 1.3 m to 1.6 m. From Fig. 6, it can be seen that the trajectory of the head in the sagittal plane is curved due to downward movement. Comparable patterns were observed in other novices.

Subsequently, the characteristics of using a video laryngoscope are described. First, the head movements of experts are presented. According to Fig. 5 (i), the displacement in the depth direction initially increases, and movement occurs within the range of 1.3 m to 1.5 m. Furthermore, as shown in Fig. 5 (ii), the displacement in the vertical direction initially decreases, and movement occurs within the range of 1.6 m to 1.7 m. From Fig. 6, it can be observed that the trajectory of the head in the sagittal plane is linear. Comparable patterns were observed in other experts.

Next, the head movements of novices are presented. According to Fig. 5 (iii), the displacement in the depth direction initially increases, and movement occurs within the range of 1.4 m to 1.5 m. In addition, as shown in Fig. 5 (iv), the displacement in the vertical direction initially decreases, and movement occurs within the range of 1.5 m to 1.6 m. From Fig. 6, it can be seen that the trajectory of the head in the sagittal plane is also linear. Comparable patterns were observed in other novices.

## Discussion

### Distance of gaze

We compared the gaze point distributions by dividing them into procedural segments and examined the characteristics associated with proficiency. To conduct detailed analysis and evaluation, four time points during the tracheal intubation procedure were defined, and three segments were delineated accordingly. The mouth-opening phase is defined as the start of the subject’s actions until the completion of mouth opening and contact with the laryngoscope using the left hand. The subsequent phase, extending from the completion of laryngeal exposure to the moment the subject touches the tracheal tube with the right hand, is defined as the laryngoscope insertion phase. The final phase, lasting until the fixation of the tracheal tube is completed, is defined as the tracheal tube insertion phase. This study focused on the laryngoscope insertion phase and the tracheal tube insertion phase, during which instruments are in motion. Gaze measurements were conducted for 15 subjects (7 experts and 8 novices) in this experiment; however, it was not possible to accurately measure the gaze data for one expert (subject 5), and there were prolonged periods during which gaze data were missing. Therefore, this subject was excluded from the analysis. First, we describe the gaze points observed when using a direct laryngoscope. As shown in the Results section, experts tended to gaze at points more distant from the mannequin, whereas novices focused on closer areas. Therefore, we calculated the distance from the subject’s viewpoint to the gaze point for quantitative analysis. Here, the gaze point refers to the intersection of the lines of sight from the right and left eyes, and the distance from the viewpoint to the gaze point is defined as the distance from the subject’s eyes to this intersection.

**Fig. 7:**
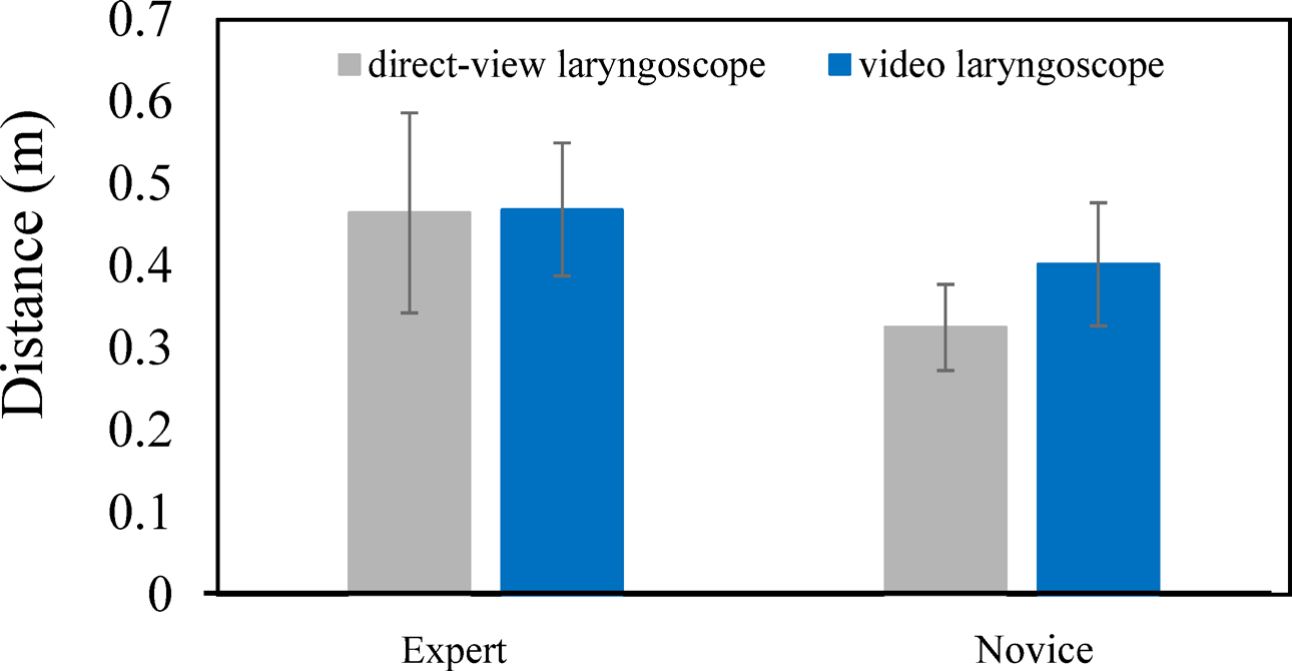
Comparison of distance from viewpoint to gaze point between experts and novices during the laryngoscope insertion phase.

**Fig. 8:**
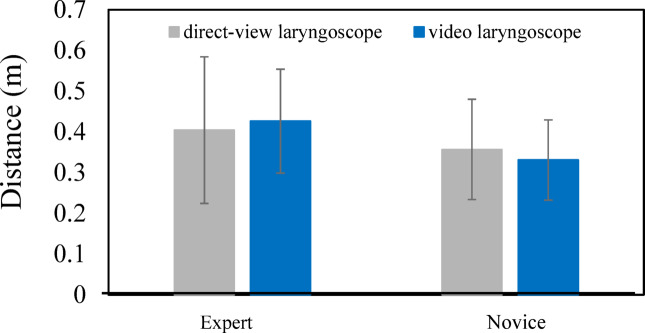
Comparison of distance from viewpoint to gaze point between experts and novices during the tube insertion phase.

According to the results for the laryngoscope insertion phase (Fig. 7), the gaze distance for experts was 0.465 $\pm0.122\mathrm{m}(\mathrm{m}\mathrm{e}\mathrm{a}\mathrm{n}\pm\mathrm{s}\mathrm{t}\mathrm{a}\mathrm{n}\mathrm{d}\mathrm{a}\mathrm{r}\mathrm{d}\mathrm{d}\mathrm{e}\mathrm{v}\mathrm{i}\mathrm{a}\mathrm{t}\mathrm{i}\mathrm{o}\mathrm{n}[\mathrm{S}\mathrm{D}\left]\right)$, whereas that for novices was 0.325 $\pm$0.052 m (mean $\pm$SD), indicating that experts tended to direct their gaze farther away. To examine whether there was a significant difference between two groups—six experts and eight novices—a t-test was performed with a significance level of 0.05. The result showed a significant difference between the two groups [t (12) = 2.690, *p* = 0.020]. These findings suggest that experts tend to adopt a broader visual perspective centered on the glottis by directing their gaze farther away, while novices focus more on their hand movements during laryngoscope manipulation and fail to achieve a comprehensive view of the glottic area. For the tracheal tube insertion phase (Fig. 8), the gaze distance for experts was 0.404 $\pm$0.180 m (mean $\pm$ SD), and that for novices was 0.357 $\pm$0.123 m (mean $\pm$ SD). Although the experts still tended to gaze farther away, the difference was smaller compared to the laryngoscope insertion phase. To examine whether there was a significant difference between two groups—six experts and eight novices—a t-test was performed with a significance level of 0.05. The result revealed no statistically significant difference between the two groups [t (12) = 0.541, *p* = 0.599]. These results show that, when a direct laryngoscope is used, differences in gaze behavior between proficiency levels are particularly evident during the laryngoscope insertion phase, and that adopting a comprehensive visual perspective during this phase is important.

Regarding the gaze points observed when using a video laryngoscope, according to Fig. 7, the distance for experts during the laryngoscope insertion phase was 0.469 $\pm$0.081 m (mean $\pm$ SD), while that for novices was 0.402 $\pm$0.075 m (mean $\pm$ SD), indicating a tendency for experts to direct their gaze farther away. However, to examine whether there was a significant difference between two groups—six experts and eight novices—a t-test was performed with a significance level of 0.05 and the result revealed no statistically significant difference between the two groups [t (12) = 1.476, *p* = 0.166]. These findings suggest that, when a video laryngoscope is used, the characteristic observed with the direct laryngoscope—namely, experts directing their gaze farther than novices—is not evident. According to Fig. 8, during the tube insertion phase, the distance for experts was 0.427 $\pm$0.127 m (mean $\pm$ SD), and that for novices was 0.331 $\pm$0.099 m (mean $\pm$ SD). Although experts again tended to gaze farther, the difference was small. To examine whether there was a significant difference between two groups—six experts and eight novices—a t-test was performed with a significance level of 0.05. The result revealed no statistically significant difference between the two groups [t (12) = 1.468, *p* = 0.168]. These findings indicate a narrowing of the gap in terms of gaze differences between experts and novices in the laryngoscope insertion phase and the tube insertion phase when using a video laryngoscope.

Taken together, these results indicate that, during the laryngoscope insertion phase, the use of a video laryngoscope appears to reduce the difference in gaze behavior between experts and novices. Furthermore, the distance from the viewpoint to the gaze point during the tube insertion phase does not differ significantly between experts and novices, regardless of whether a direct or video laryngoscope is used. Thus, during the tube insertion phase, the use of a video laryngoscope may enable novices to adopt gaze patterns similar to those of experts.

### Distance from the subject’s head to the mannequin’s mouth

**Fig. 9:**
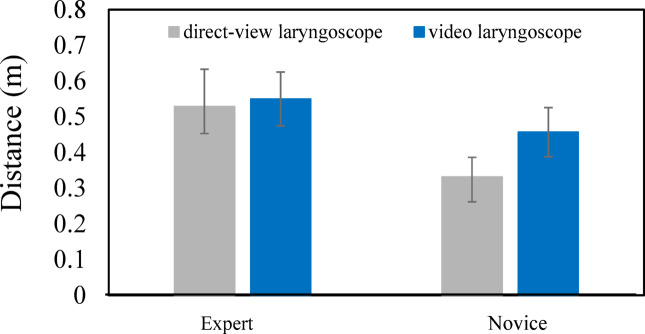
Comparison of minimum distance to the mannequin’s head between experts and novices.

We examined the characteristics of each subject’s head position, with a particular focus on the distance between the head and the mannequin’s mouth, in relation to the proficiency level. The results of the present study indicated that there may be differences in the forward and downward movement of the subject’s head depending on proficiency. Therefore, we analyzed the head positions by calculating the distance from the head to the mannequin’s mouth, and investigated how closely subjects approach the mannequin during tracheal intubation, focusing on differences associated with proficiency. Figure 9 presents the minimum distance from the head to the mannequin’s mouth. As shown in Fig. 9, when using a direct laryngoscope, the minimum distance from the head to the mannequin’s mouth was 0.529 $\pm$0.104 m (mean $\pm$ SD) for experts and 0.331 $\pm$0.055 m (mean $\pm$ SD) for novices, indicating that experts maintained a greater distance from the mannequin. A t-test conducted at a significance level of 0.05 revealed a statistically significant difference between the two groups (seven experts and eight novices) [t (13) = 4.339, *p* = 0.001]. To examine the influence of the subject’s height, a t-test was conducted to compare the heights of the expert and novice groups with a significance level of 0.05. The results showed no significant difference. These findings show that, when the subjects used a direct laryngoscope, experts tended to maintain an appropriate distance between their head and the mannequin while performing the procedure, whereas novices tended to move their head closer in an attempt to secure their line of sight. This implies that maintaining a certain distance without getting too close to the mannequin is important during the procedure.

During use of a video laryngoscope, the minimum distance from the head to the mannequin’s mouth for experts was 0.550$\pm$0.076 m (mean $\pm$ SD) and for novices was 0.457$\pm$0.069 m (mean $\pm$ SD). Although experts still maintained a greater distance, the difference between the groups was smaller than that observed with the direct laryngoscope. To examine whether there was a difference in the distance from the head to the human mannequin’s mouth when novices used a direct laryngoscope and a video laryngoscope, a t-test was performed with a significance level of 0.05. The result revealed a statistically significant difference [t (14) = 4.355, *p* = 0.001]. This shows that novices moved their head farther away when using the video laryngoscope.

Based on these observations, when they used a video laryngoscope, both experts and novices tended to maintain a greater distance between their head and the mannequin. These findings show that the use of a video laryngoscope enables novices to adopt head positioning characteristics similar to those of experts.

In summary, when novices use a direct laryngoscope, that they tend to move their head significantly, resulting in a forward-leaning posture, which in turn reduces the gaze distance. In contrast, when they use a video laryngoscope, because head movement is small, a forward-leaning posture does not occur. Therefore, it is possible for novices to perform the procedure while maintaining a gaze distance in the same manner as that of experts. In other words, for novices to perform the procedure like experts, it is important to avoid a forward-leaning posture and to keep the head tilted backward.

## Conclusion

This study simultaneously measured gaze behavior and head movement during tracheal intubation using an eye-tracking system and a motion capture system. Subjects were classified into expert and novice groups, and differences were analyzed for two types of laryngoscopes: a direct laryngoscope and a video laryngoscope. When a direct laryngoscope was used, novices exhibited pronounced forward and downward head movements and focused their gaze at close range, whereas experts maintained a backward head position and directed their gaze toward more distant areas. In contrast, the use of a video laryngoscope substantially reduced these differences. The monitor display allowed novices to obtain the necessary visual information without excessive head movement, enabling a posture and gaze strategy similar to that of experts. In other words, regardless of the type of laryngoscope, keeping the head still and stabilizing the field of view may improve execution of the tracheal intubation procedure. We believe these findings can be expected to significantly contribute to the improvement of education in the medical settings and the advancement of physicians’ medical skills.

## Supplementary Information

Below is the link to the electronic supplementary material.

Supplementary Material 1

## Data Availability

The authors declare that all experimental data supporting this study are available from the corresponding author upon reasonable request.
