# Supplementary material for: Quantitative analysis of gaze and body movement differences by proficiency in direct and video laryngoscope intubation
Source: Sci Rep. 2026 Apr 2;16:15656. doi: 10.1038/s41598-026-44432-5 (PMC13187270; doi:10.1038/s41598-026-44432-5)
Supplement: Supplementary file 1 — Supplementary Material 1 [file 41598_2026_44432_MOESM1_ESM.docx]

**Supplementary material**

Presented below are the technical specifications of the motion capture camera in this study, together with detailed information for each subject.

Specifications of motion capture cameras

| Specification | Prime13 | Prime13W |
| --- | --- | --- |
| Resolution [pixels] | 1280×1024 | 1280×1024 |
| Maximum images per second [fps] | 240 | 240 |
| Body size [mm] | 68.6(W)×68.6(H)×53.0(D) | 68.6(W)×68.6(H)×56.0(D) |
| Weight [kg] | 0.32 | 0.32 |
| Horizontal viewing angle [deg] | 56 | 82 |
| Vertical viewing angle [deg] | 46 | 70 |

| Subject | Skill level | Years of experience | Sex |
| --- | --- | --- | --- |
| 1 | Expert | 27 | Male |
| 2 | Expert | 14 | Male |
| 3 | Expert | 17 | Female |
| 4 | Expert | 40 | Female |
| 5 | Expert | 28 | Male |
| 6 | Expert | 26 | Male |
| 7 | Expert | 14 | Male |
| 8 | Novice | 1 | Male |
| 9 | Novice | 1 | Male |
| 10 | Novice | 2 | Male |
| 11 | Novice | 1 | Male |
| 12 | Novice | 1 | Female |
| 13 | Novice | 1 | Female |
| 14 | Novice | 1 | Male |
| 15 | Novice | 1 | Male |

Characteristics of each subject
